# Methane-rich saline attenuates ischemia/reperfusion injury of abdominal skin flaps in rats via regulating apoptosis level

**DOI:** 10.1186/s12893-015-0075-4

**Published:** 2015-07-31

**Authors:** Kexin Song, Mingzi Zhang, Jianqiang Hu, Yunqi Liu, Yifang Liu, Youbin Wang, Xuemei Ma

**Affiliations:** Department of Plastic Surgery, Peking Union Medical College Hospital, Beijing, China; Department of Orthopaedics, Qingdao Huangdao District Hospital of Traditional Chinese Medicine, Qingdao, Shandong China; College of Life Science and Bioengineering, Beijing University of Technology, Beijing, China

**Keywords:** Methane-rich saline, Ischemia/reperfusion injury, Skin flaps, Apoptosis

## Abstract

**Background:**

In plastic surgery, skin damage induced by ischemia/reperfusion (I/R) is a multifactorial process that often occurs. Methane gas has been reported to be a new therapeutic gas for attenuating I/R injury. In this study, we assessed the effects of methane-rich saline (MRS) in regulating apoptosis on skin flap I/R injury.

**Methods:**

Male Sprague–Dawley rats, 6–8 weeks old, were divided randomly into three groups: one sham surgery group (SH) and two surgery groups. After undergoing 6 h of I/R management of an abdominal skin flap, surgery groups were treated with physiological saline (I/R-P) or methane-rich saline (I/R-M). On the 3rd postoperative day, a laser Doppler flowmeter was used to measure flap blood supply, and hematoxylin and eosin (H&E) staining was used to observe morphological changes. TdT-mediated dUTP-X nick end labeling (TUNEL) staining was also used to observe early apoptosis and is presented as the percentage of TUNEL-positive cells. Moreover, pASK-1, pJNK, Bcl-2 and Bax were detected by immunohistochemical technology. Caspase-3 activity was also measured to evaluate the effects of MRS.

**Results:**

Compared to the I/R-P group, the flaps in the I/R-M group presented a larger survival area and better blood perfusion with less inflammatory infiltration and cell apoptosis, a higher expression of Bcl-2, a lower expression of pASK-1, pJNK and Bax, and a lower caspase-3 activity.

**Conclusion:**

According to the results, MRS attenuated I/R injury by regulating apoptosis and has the potential to be applied as a new therapy for improving skin flap survival.

## Background

Skin flap transfer is a basic plastic surgery method used for wound repair and reconstruction. Alhough surgical principles and technologies have improved significantly, flap loss caused by I/R injury after transplantation is still a serious problem to be solved [1]. During I/R injury, many cytological and morphological changes like infiltration of neutrophils happen, among which apoptosis is one of the worst outcome. The infiltration of neutrophils brings more free radicals and exacerbates the flap injury [2]. However, Burns believes that I/R injury is due to cell apoptosis [3]. Particular proteins, signal pathways and enzymes are involved leading to cell apoptosis, among which Bcl-2/Bax [4], ASK-1/JNK [5] pathway and caspase-3 [6] are common factors.

Methane is a chemical compound that is commonly used as a type of attractive fuel. In recent years, many researchers have focused on the biological characteristics of methane, showing that methane can slow intestinal transit, augment small intestinal contractile activity [7] and increase the contraction amplitude of the guinea pig ileum, although the bioactivity of methane is not a newly discovered phenomenon in medicine [8]. Moreover, Davide Roccarina also noted the role of methane in intestinal diseases. According to Roccarina’s study, it is possible to detect intestinal methane with no specific clinical relevance among 1/3 of healthy adult individuals. He also demonstrated that gas metabolism disorders and abnormal methane production are considered factors in many intestinal diseases, including colon carcinoma, diverticulosis, inflammatory bowel disease and irritable bowel syndrome [9]. In a study from 2012, Boros showed the protective effect of methane on oxidative stress and the inflammation of intestinal injury caused by ischemia and reperfusion [10]. Alhough methane has been studied in many medicinal fields, no studies have examined the protective effect of methane on skin flap injury. The flammability and explosiveness of methane gas limits its clinical use, but methane can be dissolve in physiological saline at the proper concentration without any changes to its chemical qualities. Based on all of these theories, this study aimed to test whether methane-rich saline could have any protective effect on the attenuation of I/R injury to decrease apoptosis level and improve skin flap survival in rats.

## Methods

### Animals and grouping

This animal experiment was approved by the Experimentation Ethics Committee on Animal Rights Protection of Peking Union Medical College Hospital. All procedures strictly followed the National Institutes of Health guidelines for the care and use of laboratory animals. Before and after surgery, 18 male Sprague–Dawley (SD) rats, 6–8 weeks old and weighing 280–320 g, were raised in comfortable cages at 22-25 °C with adequate food and drink.

Before the operation, 18 qualified laboratory rats were randomly divided into 3 groups: a sham surgery group (SH); a 6-h ischemia group followed by physiological saline management (I/R-P); and a 6-h ischemia group followed by methane-rich saline (MRS) management (I/R-M).

### Surgical procedure

This surgical procedure was modified based on the method reported by Küntscher [11] with some modifications [12, 13]. Laboratory rats were anesthetized intraperitoneally with 40 mg/kg pentobarbital. We designed and marked a rectangular 6 cm × 9 cm flap area on the abdomen and elevated it along a marked line based on the right superficial epigastric artery, and the left superficial epigastric artery was ligated. Flap ischemia of 6 h was induced in the I/R-P and I/R-M groups by clamping the right superficial epigastric artery with a microvascular clamp. The SH group was free from ischemia induction, but the left superficial epigastric artery was still ligated. A silicone sheet of 0.3 mm thickness was then placed between the flap and the recipient bed to prevent revascularization. Reperfusion was initiated by removing the clamp and was confirmed by the return of pulsation to the vascular arcade.

### MRS production

Methane (Beiyang Special Gas Research Institute Company, Beijing) was dissolved in 20 ml of physiological saline for 20 min at a speed of 0.2 L/min to reach a supersaturated level [12]. MRS was freshly prepared every time to ensure that the proper concentration was reached. Intrapaeritoneal injection, instead of oral way or being exposed in methane directly, was applied to make sure each animal received equal MRS or physiological saline amount in surgery groups. Fifteen minutes before and after the reperfusion was initiated, rats in the surgery groups were injected intraperitoneally with 5 ml/kg physiological saline or MRS. After that, the injection was performed once every 12 h until sacrifice.

### Evaluation of skin flap perfusion

Flap perfusion after the management above was measured on the 3rd postoperative day using a laser Doppler flowmeter (LDF, Perimed AB, Stockholm, Sweden) and laser speckle contrast analysis (LSCA, Perimed AB, Stockholm, Sweden). At an environmental temperature of 22-25 °C, the laboratory rats were anesthetized and secured on the operating table to expose the entire flap, including a part of the normal abdominal skin. Then, capillary blood flow in the dermis layer of the rats was measured by LDF. Perfusion in the necrotic and surviving abdominal flap areas was obtained automatically by delineating the specific area in the image with LSCA.

### H&E staining

**Fig. 1:**
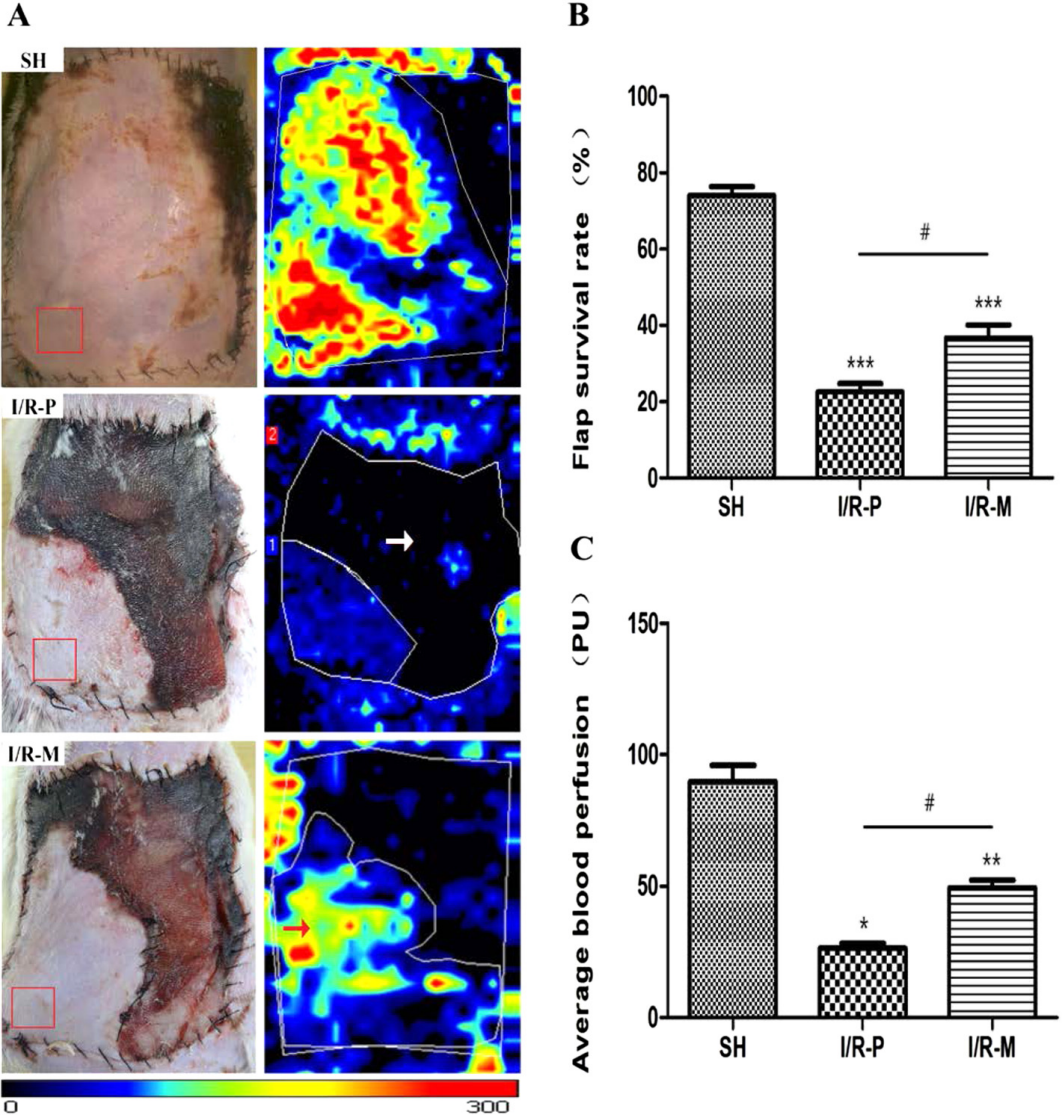
The condition of the abdominal skin flaps 72 h after I/R. The red frame represents the sampling position. The white arrow point to black zones represents the lowest blood perfusion. The red arrow point to red, yellow and the adjacent blue areas represent surviving areas with rich blood perfusion. (**a**) Representative photographs of abdominal skin flap microcirculation in the three groups are shown. (**b**) The survival rate of the total flap area. Flap survival rates were markedly higher in the I/R-M group. (**c**) The average blood perfusion of total skin flaps. The average blood perfusion of the total flap was greater in the I/R-M group than in the I/R-P group. The values are the means ± SEM; (n = 6 for each group; *p < 0.05, **p < 0.01, ***p < 0.001 versus the SH group; #*p* < 0.05 versus I/R-P group)

The laboratory rats were sacrificed by high-dose pentobarbital in the 72nd hour after reperfusion. Pieces of abdominal skin flap 1 × 1 cm^2^ in area were removed from the proximal side of the vascular axis (Fig. 1a) for H&E staining, an apoptosis assay, immunohistochemical studies and a caspase-3 activity assay. The same sampling position could avoid bias caused by different sampling position. For H&E staining, the specimens were fixed with formalin buffer. After the tissues were paraffin-embedded, sectioned, and mounted on a slide, the tissue slides were stained with H&E for histological examination.

### Apoptosis assay

Programmed cell death was assessed with the In Situ Cell Death Detection kit (POD. Roche, Basel, Switzerland) which catalytically incorporates fluorescent dUTP at 3’-OH DNA ends using a recombinant enzyme and terminal deoxynucleotidyl (TdT) transferase. The prepared paraffin-embedded sections were dewaxed and rehydrated and, then, incubated with proteinase K (20 μg/ml in 10 mM Tris/HCL, pH7.4) for 15 min at 37 °C. After these slides were rinsed with PBS (3 min, three times), they were incubated in TdT-mediated dUTP-X nick end labeling (TUNEL) reaction mixture for 60 min at 37 °C. The samples were cleared and 50 μl of Converter-POD was added for 30 min at 37 °C. After rinsing with PBS (3 min, three times), sections were developed in the dark with 100 μl DAB reaction mixture (5 μl 20 × DAB, 1 μl 30 % H2O2, 94 μl PBS) for 10 min at room temperature. Negative (by omitting TdT) and positive (by pretreating the sections with DNase I for 15 min at 37 °C) controls were used for contrast. For quantitative analysis, TUNEL-positive cells in three different slides from different skin tissues were randomly examined using a defined rectangular field area under 40× magnification. Cells were then counted under 400× magnification. The data are presented as the percentage of TUNEL-positive cells in the total number of cell nuclei per field.

### Immunohistochemical studies

Paraffin-embedded sections were routinely dewaxed and rehydrated and then incubated for 10 min with 3 % H_2_O_2_ to block endogenous catalase. Antigen retrieval was performed by heating the unstained slides in citrate buffer to 95 °C for 15 min. Incubation with normal goat serum at 37 °C for 30 min blocked nonspecific staining. Sections were placed in a humidified chamber at 37 °C for 2 h with anti-Bcl-2, anti-Bax or anti-pJNK, and anti-pASK-1 antibody (Santa Cruz Biotechnology, Dallas, Texas, USA) at a 1:50 dilution. Horseradish peroxidase-conjugated secondary antibody (ZSGB-BIO, Beijing, China) was used to mark the primary antibody. Then, the samples were flushed with PBS, stained with DAB and afterwards counterstained with hematoxylin. A brown color implied the presence of antibody bound to antigen and was detected by light microscopy with a computer-controlled digital camera and imaging software.

### Caspase-3 activity assay

Caspase-3 activity was detected using a Fluorometric Assay Kit (Bivision Research Products, MountainView, CA, USA). In brief, 50 mg of tissue was homogenized in 2× reaction buffer and incubated for 1 h at 37 °C with caspase-3 substrate (DEVD-APC)(1 mM). Substrate cleavage was measured with aspectrofluorometer at 400 nm.

### Statistical analysis

In this study, all data are reported as the mean ± standard error of the mean (SEM). Significant differences were determined via one-way analysis of variance (ANOVA). Statistical significance was set at p < 0.05. All analyses were conducted using SPSS 17.0.

## Results

### Flap survival

Necrotic flap tissues were inelastic and brown, grey or black in color on the 3rd postoperative day. In contrast, the surviving tissues were pink and elastic (Fig. 1a). Statistical results are shown in Fig. 1b. The total flap area considered to have survived was 22.59 ± 6.94 % in the I/R-P group, whereas 36.66 ± 10.90 % of the area was found to be living in the I/R-M group and 73.12 ± 6.41 % in the Sham group. In the I/R-M group, the skin flaps did not present petechiae or full-thickness necrosis like the flaps in the I/R-P group did.

### Flap perfusion

On the 3rd day after surgery, the average blood perfusion in the IR-P group was 26.25 ± 6.18 PU (ml · 100 g^−1^ · min^−1^). The average blood perfusion was 49.44 ± 9.21 PU in the I/R-M group and 85.15 ± 10.51 PU in the Sham group (Fig. 1c). The difference in average blood perfusion was obvious between the two groups (*p* < 0.05). The blood perfusion results support the vitality of the flaps with the MRS treatment.

### Histological analysis

**Fig. 2:**
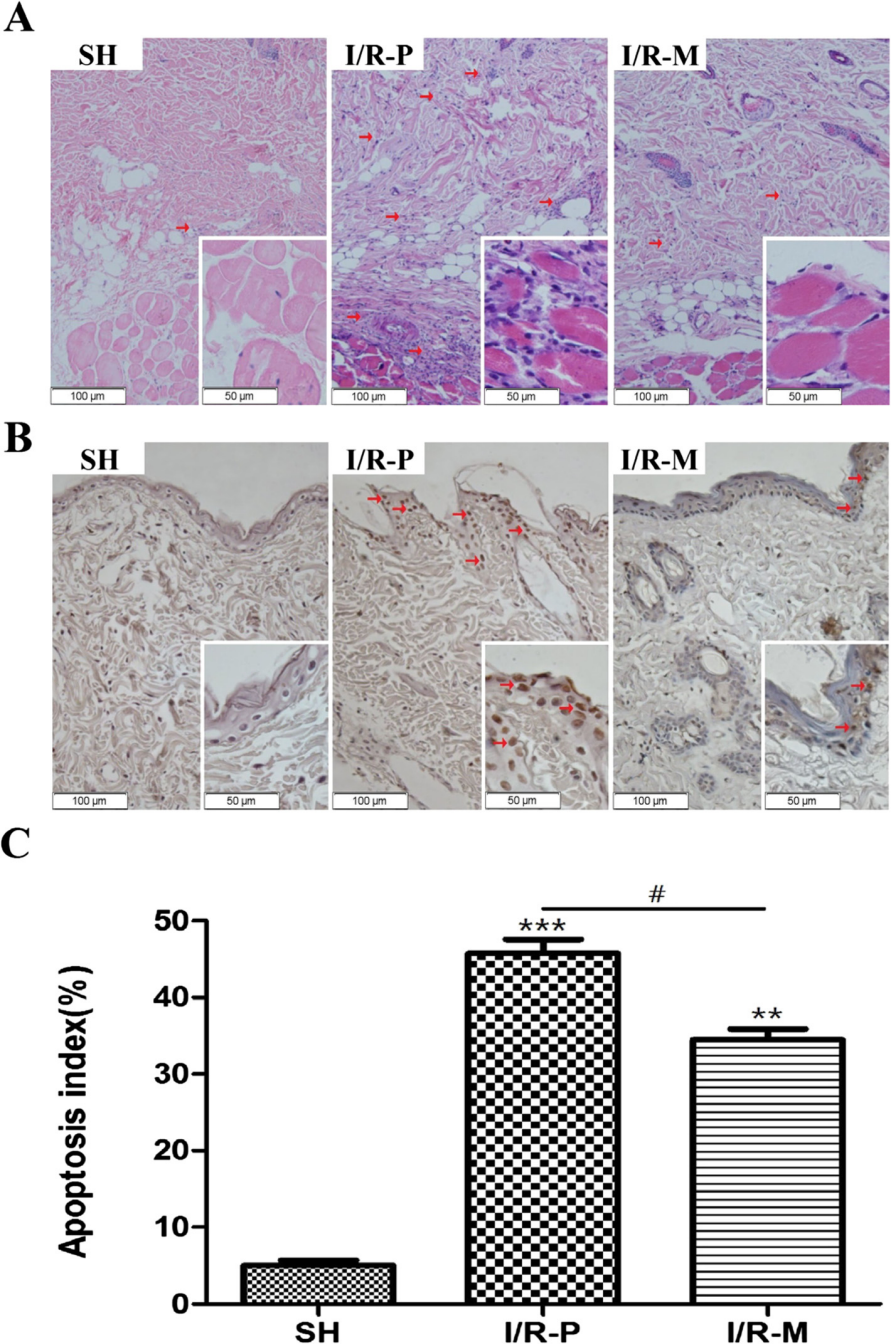
The results of H&E and TUNEL staining and the apoptosis index 72 h after I/R. (**a**) The morphological observation of flap tissue by H&E-staining in all groups. The number of infiltrated cells (red arrow) was much lower in the I/R-M group than in the I/R-P group; (big images: 200×, small image: 400×). (**b**) The evaluation of apoptotic cell death by TUNEL staining in all groups. Compared to I/R-P, MRS treatment remarkably decreased TUNEL-positive cells in the I/R-M group (brown staining indicates apoptotic cells (red arrow); big images: 200×, small image: 400×). (**c**) The apoptosis index of all groups. The data are the percentage of TUNEL-positive cells and the total cell nuclei per field, and three different slide fields from different skin tissues were counted. Values are the means ± SEM; (n = 6 for each group; **p < 0.01, ***p < 0.001 versus the SH group; #*p* < 0.05)

H&E stained tissue slices were used to assess ischemic injury (Fig. 2a). H&E staining marks inflammatory cells to reflect infiltration. Inflammatory infiltration could be observed among the surgical groups, but more inflammatory cells were found in the dermal and subcutaneous layer of the skin tissue slices from the I/R-P group. According to the results above, MRS decreased inflammatory infiltration, showing that MRS was able to attenuate the inflammatory response induced by I/R injury.

### Detection of positive apoptotic cells

TUNEL-positive cells were mainly stained brown as shown in Fig. 2b. Few apoptotic cells (5.08 ± 1.90 %) were observed in the Sham group. Compared to the I/R-P group (45.83 ± 5.61 %), the MRS management of skin flaps clearly decreased the percentage of apoptotic cells (34.47 ± 4.46 %) (Fig. 2c).

### Bcl-2, Bax, pASK-1 and pJNK Immunohistochemical studies

**Fig. 3:**
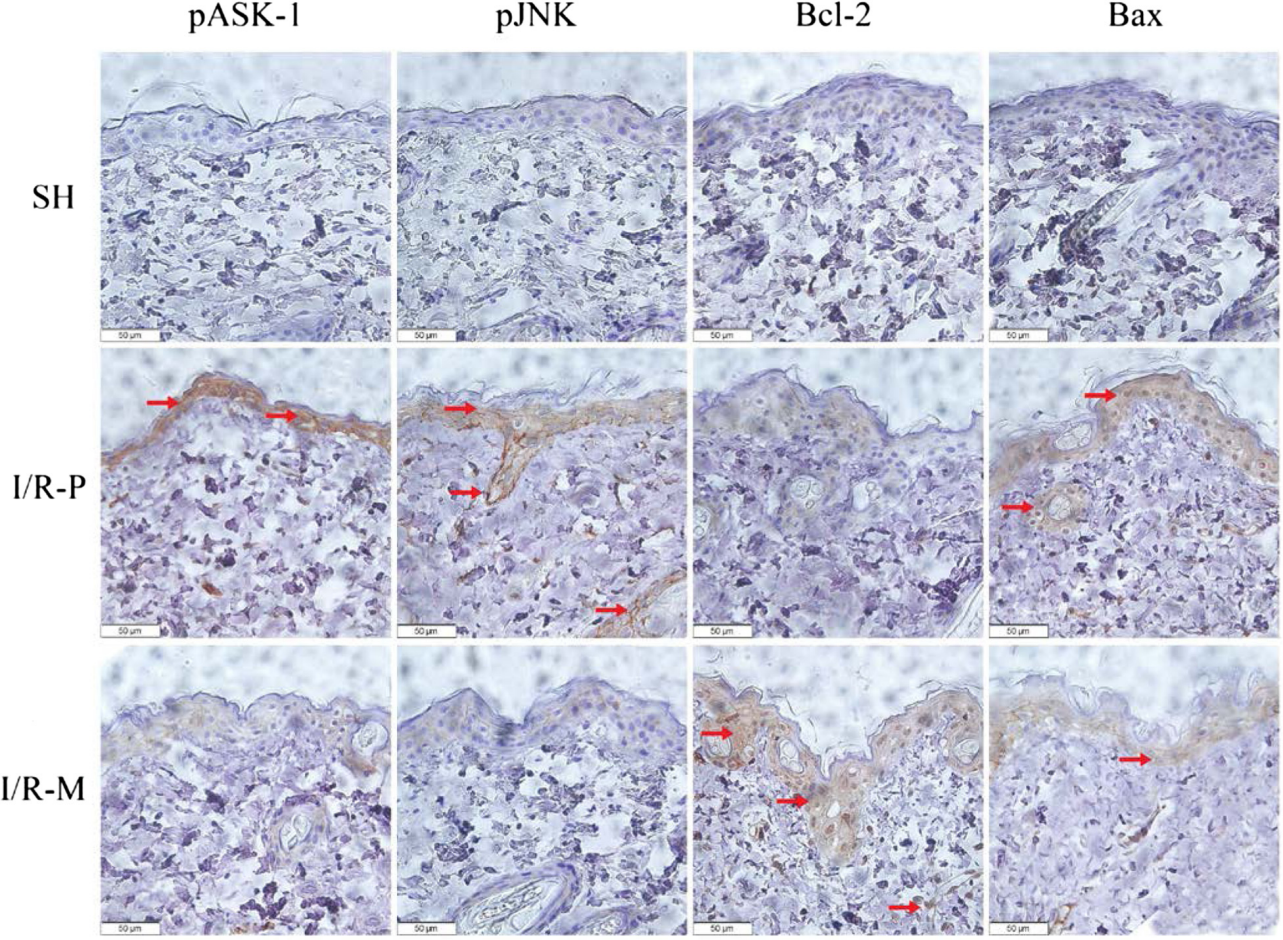
An analysis of apoptotic factors 72 h after I/R. Representative micrographs (400×) of flap tissue immunohistochemistry for pASK-1, pJNK, Bcl-2 and Bax in all groups are presented above. Brown staining indicates positive expression areas, and the shade of color represents the expression level of the target protein. The levels of pASK-1, pJNK and Bax were lower in the I/R-M group than in the I/R-P group, with Bcl-2 displaying the opposite pattern

Immunohistochemical studies reflect the expression of target proteins, such as Bcl-2, Bax, pASK-1 and pJNK which have been shown to be key proteins in apoptosis. The results showed that the expression of Bcl-2 was much higher in the I/R-M group, whereas the expression of Bax, pASK-1 and pJNK was lower in the I/R-M group than in the I/R-P group (Fig. 3).

### Caspase-3 activity

**Fig. 4:**
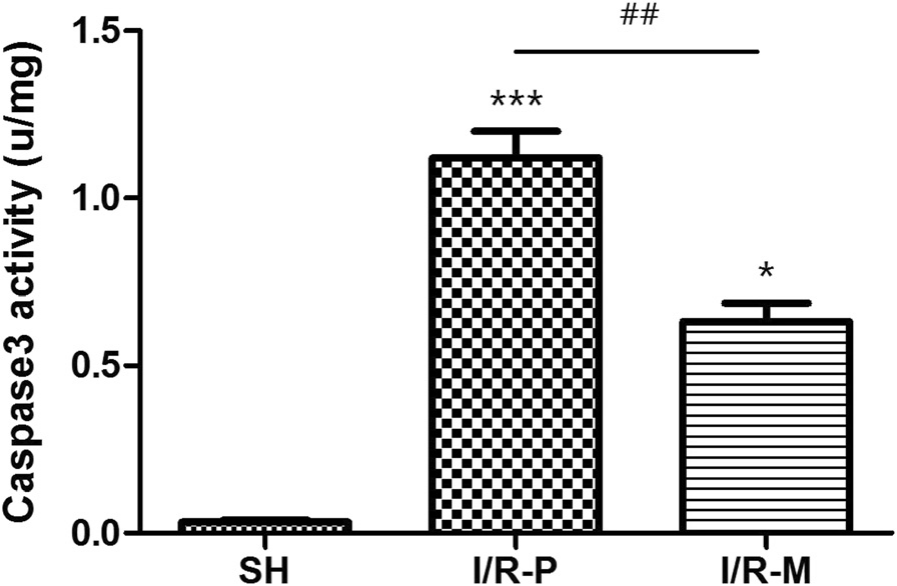
Caspase-3 activity in all groups 72 h after I/R. Compared to the I/R-P group, caspase-3 activity was significantly inhibited in the I/R-M group. Values are the means ± SEM;(*n* = 6, *p < 0.05, ***p < 0.001 versus the SH group; ##*p* < 0.01)

Caspase-3 activity was clearly upregulated into the reperfusion process in the I/R-P group. The rats subjected to skin flap I/R followed by MRS (0.63 ± 0.17) presented decreased in caspase-3 activity compared with the I/R-P group (1.12 ± 0.25) rats (Fig. 4). In Sham group, caspase-3 activity was (0.032 ± 0.09). These data revealed that methane suppressed cell apoptosis via attenuated caspase-3 activity.

## Discussion

In the plastic surgery field, flap complications often occur in flap transfer operations. Although many strategies, including pharmaceutical agents, preconditioning and postconditioning, have been investigated to prevent such unwanted outcomes, the total loss of skin flaps in microsurgical transplantation is approximately 1 % to 5 % in experienced hands, partial flap loss is 7 % to 20 % of free flaps and 20 % to 30 % of pedicled flaps [12]. There are many reasons for flap failure, among which I/R injury is an important one. Neutrophil infiltration is a classic characteristic of acute inflammation during I/R injury, and has been shown to worsen reperfusion injury by leukocyte activation [2]. In addition, apoptosis is induced by I/R process in skin-flap tissue and leads to cell shrinkage, nuclear condensation and cell death [3], which is one of the worst outcomes for a skin flap.

Many researchers have agreed that methane is a therapeutic agent. According to the Occupational Safety and Health Administration of United States, methane has been regarded as a simple asphyxiant that is intrinsically nontoxic. Many antioxidants have limited therapeutic utility because of the impermeability of membranes [14]. Methane, which is a type of gas with small molecules, has the proper distribution characteristics to penetrate membranes and diffuse into organelles [7, 15]. In addition, in Boros [10] and Varga’s study [16], data demonstrate the anti-inflammatory profile of methane during I/R process. But there is no study about methane and skin flap research especially on apoptosis aspect. Therefore, our study focused on the protective effects of methane in regulating apoptosis level on I/R injury to rat abdominal skin flaps.

In our study, an abdominal-island skin-flap ischemia and reperfusion model induced by ligating the left superficial epigastric artery was used to investigate cellular and molecular changes after MRS treatment. According to professor Sun’s study [17], methane is more effective in attenuating hepatic ischemia-reperfusion injury through injection than inhalation. Like hydrogen, methane is small molecules, which means most methane molecules could be released though interstitial space to the external besides the absorbed ones. In this case, the injection of MRS was performed once every 12 h, which could not only maintain the effect of MRS but also avoid the intra-abdominal infection induced by repeated intraperitoneal injection. After 6 h of ischemia and reperfusion, I/R injury was observed as large necrotic areas and low levels of flap blood perfusion. Intraperitoneal injection of MRS improved flap blood perfusion to increase the flap survival rate and attenuated cell apoptosis level and infiltration of inflammatory cells.

The mechanisms of cell apoptosis induced by I/R injury is still complicated with many signal pathways, proteins and enzymes involved. Mitochondrial reactive oxygen species generated by TNF-α can oxidize the reduced thioredoxin-apoptosis signal-regulating kinase 1 complex (Trx(SH)_2_-ASK-1), then activate ASK-1 and its downstream stress signaling targets, such as JNK [18–21], and initiate the apoptosis procedure. Many proteins regulate the apoptosis process, among which one pair of proteins is paramount, the anti-apoptotic protein Bcl-2 and the pro-apoptotic protein Bax. These two proteins can combine to form a heterodimer and its ratio determines the fate of cells [22]. Caspase-3 plays an irreplaceable role in facilitating the apoptosis process. Caspase-3 can be activated by many factors, such as reactive oxygen species and the low expression of Bcl-2. Activated caspase-3 can cut PARP (poly(ADP-ribose)polymerase) and increase the activity of Ca^2+^/Mg^2+^-dependant endonuclease to destroy DNA molecules [23]. Most studies explain the mechanisms of protective effects in terms of flap survival area, histological examination and other aspects. However, our study focused not only on skin flap survival evaluation, but it also analyzed the results from the perspective of apoptosis, which offers a new approach to explaining the protective effects of MRS against I/R injury.

TUNEL staining and the expression of the Bcl-2, Bax, pASK-1 and pJNK proteins reflect the apoptotic condition. Moreover, caspase-3 can be activated by reactive oxygen species and the low expression of Bcl-2 during I/R injury and promote the apoptosis. In our results, Bcl-2 was expressed more highly in the I/R-M group than in the I/R-P group. Bcl-2 was the first anti-apoptosis gene found and is a proto-oncogene. Bcl-2, known as a mitochondrial anchoring protein, may prevent a reputed reactive-oxygen-species-induced step during apoptosis by working like an antioxidant partner and regulating the permeability of the mitochondrial membrane [24]. During apoptosis procedure, Bcl-2 inhibits Bax relocalization, mitochondrial membrane depolarization, nuclear fragmentation, caspase activation and cytochrome-c release [25, 26]. The decline of active JNK and ASK-1 may have caused the Bcl-2 upregulation phenomenon in the I/R-M group. The anti-apoptotic effects of Bcl-2 can be inhibited by enhanced Bcl-2 phosphorylation under certain specific stimuli [27]. Methane may improve the level of Bcl-2 indirectly through an unknown pathway. In the I/R-P group, Bax in the extracellular matrix formed homodimers and anchored on the outer mitochondrial membrane to build channels that released cytochrome-c and consequently induced apoptosis [28]. Compared with the I/R-P group, the expression level of Bcl-2 was higher and Bax was markedly lower in the I/R-M group, so the formation of Bax homodimer could be inhibited by a heterodimer of Bcl-2 and Bax [29]. The caspase-3 activity results also indicated that apoptosis was clearly attenuated in the I/R-M group. Compared with former studies of apoptosis, our findings show similar results. According to the immunohistochemical studies, the protective effect of MRS was revealed by the decreased expression levels of activated ASK-1, JNK and Bax, and the increased expression of Bcl-2.

In addition to methane, many other gases have been discovered to have therapeutic effects, such as hydrogen and hydrogen sulfide [30, 31]. Their protective effects are accepted and have been shown in animal models [31–33]. However, the application of therapeutic gases in clinical work is still difficult because of their explosive and flammable properties and the difficulty of transportation. However, these types of gases can dissolve in physiological saline at the proper concentration without any changes in their chemical properties. Thus, normal saline rich in therapeutic gas may be prepared as an injection which would be more practical and safer to use. Compared with hydrogen and hydrogen sulfide, methane is much cheaper and more easily produced, and moreover, methane can reach a higher concentration when dissolved in physiological saline [34]. However, methane has been received much attention recently as a contributor to climate change and global warming. Therefore, the recycling of methane should be considered. Moreover, methane may be regarded as a factor in some gastrointestinal disorders [9]. Therefore, much more investigation is still needed before methane can be used in gastrointestinal diseases [15].

## Conclusion

According to the results of our study, compared with the I/R-P group, the skin flap survival rate was improved and the cell apoptotic condition was notably attenuated in the I/R-M group. Therefore, we postulate that MRS could be a novel anti-apoptotic agent with a promising role in decreasing I/R injury in rat abdominal skin flaps.
